# Reply to: Silica is unlikely to be soluble in upper crustal carbonatite melts

**DOI:** 10.1038/s41467-023-35841-5

**Published:** 2023-02-21

**Authors:** Jasper Berndt, Stephan Klemme

**Affiliations:** Institut für Mineralogie, Westfälische Wilhelms-Universität Münster, Corrensstraße 24, 48149 Münster, Germany

**Keywords:** Geochemistry, Geology

**replying to** M Anenburg & T Guzmics *Nature Communications* 10.1038/s41467-023-35840-6 (2023)

In a recent paper, we present evidence for immiscible Ca- and Si-rich carbonatite and phonolite melts that were found in hauyne-hosted melt inclusions from the Laacher See volcano in the Eifel, Germany^1^. In their comment, Anenburg and Guzmics (hereafter A&G) describe one experiment performed to assess whether they could replicate the observed immiscible carbonatite liquid from the Laacher See volcano^1^. Their experimental run product shows that the starting material did not melt, that the run was quite obviously subsolidus and, consequently, contained no carbonatitic liquids. However, the absence of melt can be explained by the experimental techniques A&G used. We were surprised to see that the experiment was run in a piston cylinder apparatus with uncalibrated assemblies, very similar to those used in a previous experimental study^2^. Piston-Cylinder assemblies suffer from large friction, especially at low run temperatures, and this is the reason why most modern experimental laboratories use these assemblies only at run temperatures of well above 1000 °C, and only after careful calibration (e.g., ref. ^3^). There is a plethora of information about different piston cylinder assemblies and the associated problems with friction in the literature (e.g., refs. ^4,5^). The use of uncalibrated piston-cylinder assemblies without friction correction led A&G to substantially underestimate the actual pressure of their run. As the solidus and the liquidus of a volatile element-rich system critically depend on pressure, we are not surprised that the single experiment of A&G did not melt at all. However, we agree with A&G that experiments are needed to map out the new immiscibility gap between phonolitic melts and SiO_2_-rich Ca-carbonatites^1^. However, these are difficult experiments that need to be well documented and they need to be carried in a well-calibrated high-pressure apparatus.

A&G claim in their comment that carbonatite melts at crustal pressures and temperatures cannot contain more than 5% SiO_2_. However, several experimental studies clearly show that crustal carbonatite melts can easily contain between ≥12 (e.g., refs. ^6,7^) and 30 wt.% of SiO_2_ (e.g., refs. ^8,9^). It is quite obvious that these experimental results, and our observations at the Laacher See volcano^1^, do not agree with a simplistic model (e.g., refs. ^2,10,11^) that aims to explain the origin of Ca-rich carbonatites, in essence, by loss of Na (and gain of Si amongst other elements) from a parental Na-rich carbonatite melt to the surrounding wall rocks. Whilst we do not claim that this process may not occur in nature at all, our findings simply imply that the model may not be applicable to all carbonatites and our observations clearly show that SiO_2_-rich carbonatite melts are stable at crustal conditions. However, more research on these melts is needed and future studies may show that the validity of previous genetic models of crustal carbonatites need to be reconsidered.

Furthermore, A&G speculate that our reported carbonatite analyses suffered from analytical artefacts, namely alkali-loss, mixed analyses of phonolite-carbonatite melts and hauyne, and droplets of silicate in the carbonatite melt.

As to the droplets, *some* of the melt inclusions reported by ref. ^1^ show the incomplete physical separation of silicate and carbonate melts as they represent early stages of liquid immiscibility. However, A&G falsely conclude that *all* carbonatite melts contain such silicate droplets and that all analyses could be contaminated. This is clearly not the case. The high z-contrast between silicate and carbonatite melt allows accurate identification of carbonatite (Lc) droplets in silicate glass (Ls) and vice versa. Consequently, we strictly avoided analysing “contaminated” carbonatite blebs and hence there was no need for corrections of any kind. The existence of pure, silicate droplet-free carbonatite melt blebs as identified by high-resolution backscattered electron imaging is exemplarily demonstrated by high-resolution FE-microprobe maps confirming the absence of Ls droplets (melt inclusions (i) and (ii) in Fig. 1b). Melt inclusion (iii) in Fig. 1 depicts an example of a carbonate bleb that contains silicate melt droplets but, as mentioned before, those blebs were *not* analysed.

**Fig. 1:**
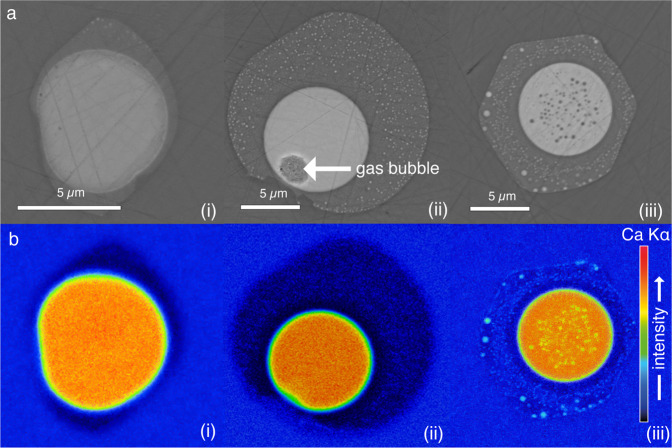
Back scattered electron images of selected melt inclusions. BSE (**a**) and X-ray intensity maps of Ca K$\alpha$ (**b**) of three exemplary melt inclusions. Melt inclusions (i) and (ii) contain no silicate melt droplets (Ls) in the immiscible carbonatite (Lc), neither in BSE images nor in X-ray maps. The melt inclusion (iii) does contain small silicate blebs in the carbonatite. Only melt inclusions without silicate blebs were used for quantitative analyses.

Given the small size, most of the carbonatite blebs were analysed using EPMA spot sizes of 2–5 µm, short counting times (5 s) and a beam current of 10 nA. As migration of alkalis during analysis can indeed be a problem, we recorded the peak signals of Na and K, as well as Si and Ca, on multiple carbonatite blebs as a function of time and spot size. Figure 2 shows a significant loss of Na and to a lesser extent K over a beam exposure time of 40 s, being expectedly more pronounced with decreasing spot size. However, as we always used only 5 s peak counting times for quantification, the alkali loss is much less significant, in the range from ∼10 to 16% relative, depending on the spot size. Thus, we expect our reported alkali concentration to be correct within <20% relative of the true values.

**Fig. 2:**
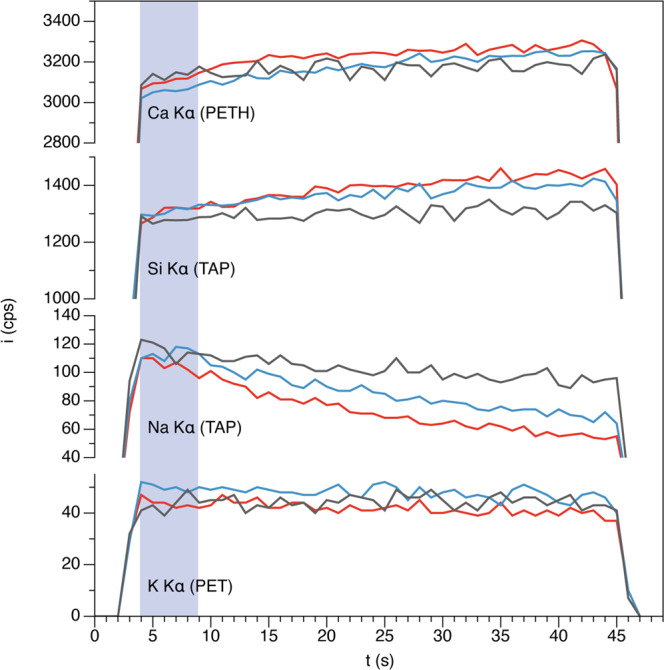
X-ray intensities (i in counts per second (cps)) of Ca, Si, Na, and K on Lc plotted against time using spot sizes of 5 μm (grey), 2 μm (blue), and 1 μm (red). Also shown is the 5 s EPMA integration time (grey area) used for the quantification of Lc and Ls.

Although A&G do not report quantitative analyses of the phases identified in their single subsolidus experiment, they claim to have found hauyne and raise the possibility that hauyne might have also contributed to our carbonatite analyses. We want to stress that we never found any hauyne inclusion in neither the carbonatite nor the silicate part of the melt inclusions. Nevertheless, as hauyne is the host mineral of the melt inclusions care must be taken to avoid mixed analyses. The hauyne contains around 12 wt.% SO_3_ and a hauyne contribution of 10% to the overall carbonatite signal would result in SO_3_-contents of >1.6 wt.%, clearly above the Lc average SO_3_ concentration of 0.38 wt.%^1^. A significant contribution of hauyne to the carbonatite analyses can thus be ruled out.

Finally, A&G criticise the fact that the immiscible carbonatite and phonolite melts are very different from previously reported immiscible melt pairs, but this is exactly what makes our new findings so exciting and we believe the immiscible melts from the Laacher See volcano will spur further research into the origin of carbonatites.

## Data Availability

The data supporting the findings of this study are available from the authors
